# Ioduret of Sulphur in Porrigo

**Published:** 1842-05

**Authors:** 


*Ioduret of Sulphur in Porrigo.*—Dr. Wm. Davidson states that he has found no remedy so efficacious in the treatment of porrigo, as the ioduret of sulphur, having repeatedly succeeded in curing the patient permanently with it after a long and fruitless trial with other remedies. His method of treatment is the following:

The head is first well washed with soap and water, the hair is then cut as short as possible with scissors, a poultice is applied, and continued for a day or two if necessary, to soften the crusts, which being removed as thoroughly as possible, the hair is closely shaved. In general, the ointment is not applied until the head has been shaved, but if pediculi be present, it is employed from the commencement, in order speedily to extinguish these vermin. The proportion of ioduret of sulphur employed has varied from 20 to 40 grains to one ounce of axunge; but, in general, the latter quantity may be safely used from the beginning, unless there be some unusual inflammatory action present; for it seldom excites any particular pain or irritation. As a general rule, tho daily application of the ointment will be sufficient, but in some cases, it is advisable to use it twice a day in order to facilitate the cure.

Alteratives, or any particular internal treatment, have rarely been resorted to, when the general health was tolerably good, Laxatives have occasionally been prescribed, and a mild farinaceous or milk diet.—*London and Edinburgh Monthly Journ. of Med. Sci.* Dec. 1841.

